# Editorial Notes

**Published:** 1935

**Authors:** 


### Editorial Notes

**The Silver Jubilee.**

THE Silver Jubilee of His Majesty King George the Fifth was the occasion of a wonderful outburst of loyal affection throughout the Kingdom and the Empire. The crowds that lined the London streets as the King and Queen drove in procession to the Thanksgiving Service at St. Paul's on 6th May manifested by their orderliness no less than by their cheers a devotion and gratitude to our Sovereign and his Queen which were unmistakable. Not only in London, but in every town and village throughout the King's dominions the same feelings were demonstrated. In his reign of twenty-five years our King has shared with his people many vicissitudes of fortune. These years have been fraught with perils and difficulties as well as marked by victory and times of prosperity ; throughout them all the wisdom and kindness of heart of the King have been observed and appreciated by his subjects. His dangerous accident during the War and his serious illness in 1928 served to emphasize the manner in which the whole Empire depended on him and looked to him for support. Doubtless our anxieties for his health on these two occasions served to enhance our esteem for his qualities as a ruler.

Three out of the five visits paid to Bristol by their Majesties during the King's reign have been events of much importance to the medical profession here. On 28th June, 1912, the King and Queen opened the

King Edward VII Memorial Infirmary ; in addition to granting permission for two wards to be named the " King George " and " Queen Mary " wards in honour of their visit a third ward was named after their daughter, H.R.H. Princess Mary (Princess Royal). This was the outcome of a spontaneous and unexpected request by their Majesties, which won all hearts as much by the manner in which it was made as by the honour it conferred. A year later, on 4th July, 1913, the King re-visited Bristol on the occasion of the Royal Show on the Downs. On his way His Majesty stopped at the Victoria Rooms, where the King Edward VII Memorial Statue had just been unveiled.

The King and Queen visited Bristol twice during the War. In September, 1915, they made a private visit to the Second Southern General Hospital, which during the War occupied the King Edward VII Memorial Infirmary and the whole of the then newly-finished Southmead Hospital. Those who had the honour of accompanying their Majesties round the wards will never forget the kind way in which they talked to the patients nor the surprise of the patients at the ease they felt in their conversations.

The second visit during the War took place in November, 1917, and was of a more public character. On that occasion their Majesties visited various factories and met munition workers and trade union leaders at the Council House and in the afternoon the King held an investiture on Durdham Down.

The last visit of the King and Queen was made in 1925, when the new University buildings were opened by His Majesty. The members of the medical faculty were justly proud to see the achievement of a project which Doctors Carrick and James Cowles Prichard were the first to contemplate in 1833.

Before he ascended the throne King George had visited Bristol, when as Prince and Princess of Wales he and Queen Mary came in 1902 to cut the first sod of the Royal Edward Dock at Avonmouth.

It is not only the recollection of what these royal visits have meant to Bristol that fills us with gratitude, it is rather the contemplation of twenty-five years of unremitting service to their people that makes us rejoice at the opportunity of acknowledging with humble loyalty all that we owe to our King and Queen. There has been something in the personal relations between our Sovereign Lord and his subjects that has justified John Buchan in replacing the term "The King's Majesty" by "the King's Grace."

**Industrial Medicine and Traumatic Surgery.**

THE Clinical Congress of the American College of Surgeons held at Boston in October, 1934, provided some very interesting topics for discussion, and has directed attention to matters which Bristol will do well to consider. The great work of Böhler's Clinic* in Vienna is well known to most of our readers, but there is some danger lest his work on fractures should overshadow the other departments of surgery and medicine which are worthy of imitation. From the report of the Congress of the American College of Surgeons it is evident that the lessons of Böhler's Clinic are being digested and turned to good account in the United States. There is no reason for Bristol to be reluctant to explore new territory, this would accord ill with our traditional independence and our

* See Review of Dr. Böhler's work, *The Treatment of Fractures*, on page 129.

historic impulses. The American College of Surgeons considers that a minimum standard for medical service in industry has been perfected, and clinics which specialize in industrial medicine and traumatic surgery are under survey by the College to consider which are equipped to give proper service. This service is not designed for dealing with accidents only, because illness has been found to cause eight times as much absenteeism as accidents. One of the Insurance Company Managers taking part in the discussion asked, " Why are we not getting better end-results in our industrial injuries ? " The answer appears to be that the principles of war-surgery are only slowly being applied to the accidents of civil life and industry. At the present time Vienna alone can claim a completely modern service for industrial medicine and traumatic surgery. Kanavel advocated, at the American Congress, that " employers and insurance companies should be educated as to the financial saving by prompt treatment and early hospitalization in cases of doubt."

In Bristol the demand is in our opinion less likely to come from employers and insurance companies than from the work-people themselves. The debt which our hospitals owe to " Works - Governors " is not to be measured by money contributions only, though these have been in truth generous enough. The addition to the Managing Boards of " Works-Governors " has had a vitalizing effect. Men and women of wealth, large employers, and ardent social workers have in the past made and maintained our voluntary hospitals, but these with all their zeal and generosity have always lacked some of the inside knowledge, the view-point of the patient, which "Works-Governors " bring to our hospital committee meetings.

Every now and then some parts of our medical practice become either out of date or thoroughly bad. In every department of our hospital practice we must look to it that what we observe praiseworthy in others we shall carefully imitate and what may appear defective we shall in ourselves amend. The department which at the present time seems capable of amendment is that which is known as the Casualty Department, where injuries, accidents, cases of sudden or urgent sickness are first seen. The Casualty Department of a hospital is usually, as *The Lancet* observes, "in charge of an officer without mature experience and not necessarily with any special skill in accident work." We do not, as yet, resort to *early hospitalization in cases of doubt.*

Are we satisfied that the practice, not the study, of medicine and surgery is, in this branch, equal to the best standards? Or to put the question in the words of the inspired author of *Rab and His Friends*, is our sagacity up to our science?

